# Strain induced band inversion and topological phase transition in methyl-decorated stanene film

**DOI:** 10.1038/s41598-017-17336-8

**Published:** 2017-12-06

**Authors:** Dongchao Wang, Li Chen, Hongmei Liu, Changmin Shi, Xiaoli Wang, Guangliang Cui, Pinhua Zhang, Yeqing Chen

**Affiliations:** Institute of Condensed Matter Physics, Linyi University, Shandong, 276000 China

## Abstract

The researches for new quantum spin Hall (QSH) insulators with large bulk energy gap are of much significance for their practical applications at room temperature in electronic devices with low-energy consumption. By means of first-principles calculations, we proposed that methyl-decorated stanene (SnCH_3_) film can be tuned into QSH insulator under critical tensile strain of 6%. The nonzero topological invariant and helical edge states further confirm the nontrivial nature in stretched SnCH_3_ film. The topological phase transition originates from the *s*-*p*_*xy*_ type band inversion at the Γ point with the strain increased. The spin-orbital coupling (SOC) induces a large band gap of ~0.24 eV, indicating that SnCH_3_ film under strain is a quite promising material to achieve QSH effect. The proper substrate, *h*-BN, finally is presented to support the SnCH_3_ film with nontrivial topology preserved.

## Introduction

Two dimensional topological insulators (2D TIs)^1–6^, also known as quantum spin Hall (QSH) insulators, have attracted extensive attention in condensed matter physics and material science because of their novel and unique properties. QSH insulator can be characterized by insulating bulk states and metallic edge states. Such edge states are protected against nonmagnetic disorder by time-reversal invariant. This newly discovered class of materials holds a promising potential for applications in quantum computation and spintronics.

Group-IV elemental layered films have stimulated enormous research due to their extraordinary physical, electronic and topological properties, since the graphene was successfully exfoliated^7–10^. For example, graphene exhibits extra high electron mobility, which is very suitable for ultra-fast switching. Silicene and germanene were identified as QSH insulator^11^. However, the bulk band gaps in them are too small because of weak spin-orbital coupling (SOC). 2D tin film, namely stanene, has recently been theoretically predicted to be QSH insulator^12,13^, which seems to possess the largest nontrivial gap (0.1 eV) which could be achieved for 2D group IV films. Although stanene has been synthesized on Bi_2_Te_3_ (111) substrate by experiment^14^, stanene on Bi_2_Te_3_ (111) displays metallic character due to lattice mismatch of 6.4%, which hinders the application of stanene in electronic devices. Up to date, there are no stable free-standing species of stanene that are experimentally accessible to perform QSH effect. Chemical adsorption is one of most commonly used methods to stabilize 2D thin films, enlarge band gap and modulate their electronic property^15–19^. Very recently, we notice that methyl-substituted germanene, namely GeCH_3_, was successfully produced, in which the thermal stability is strongly enhanced^20^. However, GeCH_3_ was proposed to be trivial insulator, which would become nontrivial phase under tensile strain of 12%^21^. Such large strain, particularly biaxial tensile strain, is very challenging to implement for 2D materials. Motivated by this point, methyl may promote stanene to be one of good candidates to achieve QSH effect due to strong SOC strength in Sn atoms.

In present work, we achieve a QSH insulator with large energy gap in SnCH_3_ film via in-plane tensile strain by means of first-principles calculation. At a critical value of tensile strain of 6%, the topological phase transition from trivial to nontrivial insulator occurs due to band inversion at the Γ point. Its QSH states are confirmed by nonzero topological invariant Z_2_ and topologically protected helical edge states established in nanoribbon. For practical application in electronic devices, we proposed *h*-BN sheet as suitable substrate to support SnCH_3_ film with nontrivial topology maintained because of suitable lattice matching. Our results show that stretched SnCH_3_ is an ideal candidate to realize QSH effect and quite promising for application in spintronics.

## Computational details

The first-principles calculations based on density-functional theory (DFT) were performed by the Vienna ab initio simulation package (VASP)^22^, using the projector-augmented-wave potential^23,24^. The Perdew-Burke-Ernzerhof (PBE)^25^ generalized gradient approximation (GGA) was used to describe the exchange-correlation potential. The kinetic energy cutoff is set to 500 eV and the convergence threshold for energy is 10^-6^ eV. All atom positions are fully optimized until the forces on each atom is less than 10^–3^ eV/Å. The Brillouin zone integration is performed with a 17 × 17 × 1 k-mesh for geometry optimization and self-consistent calculations. To simulate isolated thin films, a sufficiently large vacuum space of 20 Å is used to rule out any interactions between the neighboring films. The SOC is included in self-consistent electronic structure calculations. Phonon spectrum is calculated for a 5 × 5 × 1 supercell by density functional perturbation theory using VASP and PHONOPY^26^.

## Results and Discussion

**Figure 1:**
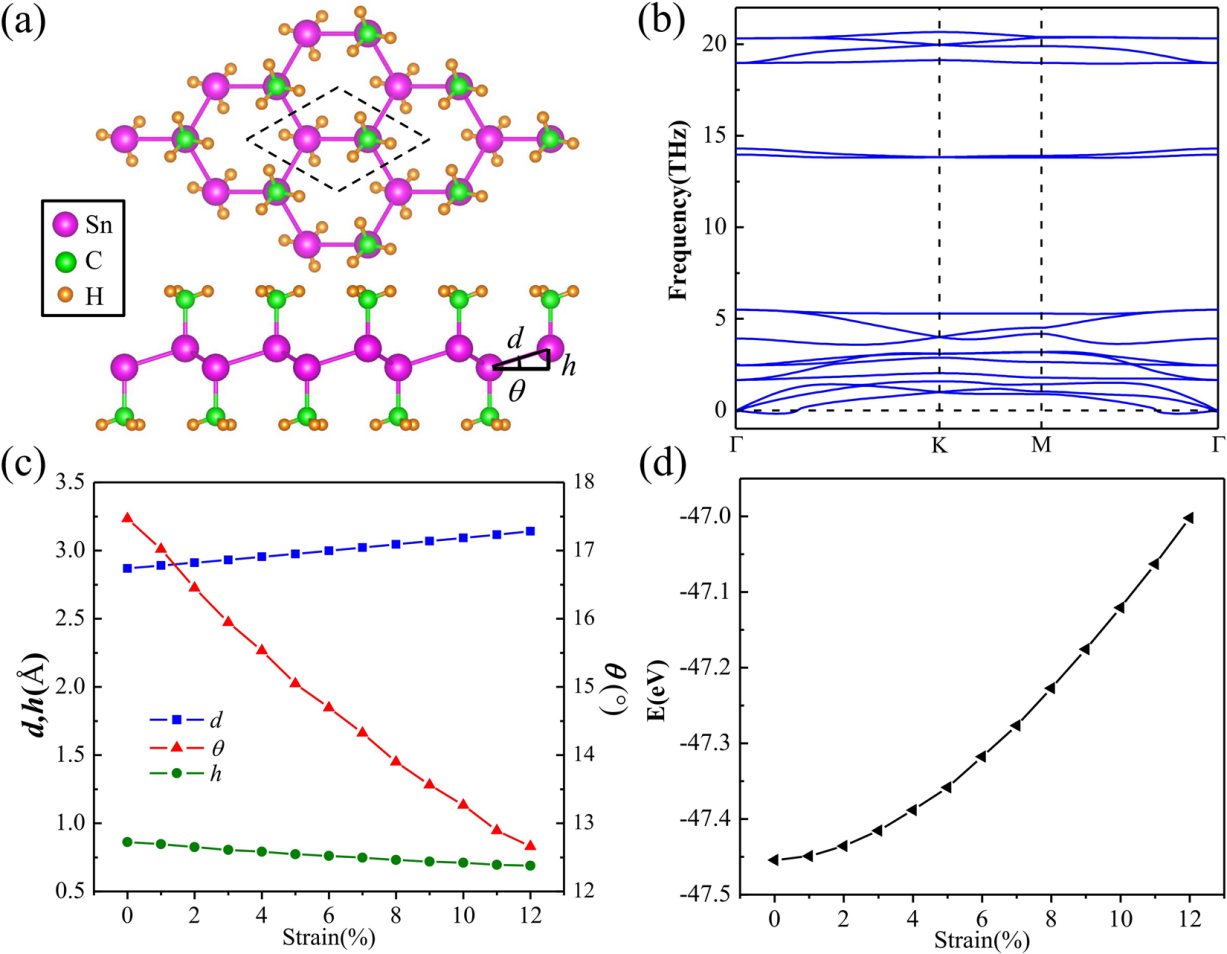
(**a**) Top and side views of atomic structure of SnCH_3_ film. (**b**) Corresponding phonon spectrum of SnCH_3_ film in (a). (**c**) The evolutions of the buckling height (*h*), Sn-Sn bond length (*d*) and buckling angle (*θ*) indicated in (**a**) under tensile strain. (**d**) The variation of total energy as a function of tensile strain.

Figure 1(a) presents the geometric structure of SnCH_3_ film from top and side views. The CH_3_ groups are adsorbed on Sn atoms on both sides of stanene in an alternating manner. There are ten atoms per unit cell, including two Sn atoms, two C atoms and six H atoms. Via geometric relaxation, the lattice parameters can be obtained. The lattice constant (*a*) and Sn-Sn bond length (*d*) are 4.74 Å and 2.87 Å, respectively, which are slightly expanded than the case of pure stanene. The buckling height (*h*), Sn-C bond and C-H bond length are 0.86 Å, 2.20 Å and 1.10 Å, respectively. The hexagonal lattice and structural inversion symmetry are still maintained.

The kinetic stability of SnCH_3_ film is confirmed by calculating the phonon spectrum. There is only minor imaginary frequency appearing near the Γ point as observed in Fig. 1(b). In fact, such small imaginary frequency is a common issue in first-principles calculation for 2D materials, such as germanene, stanene^8,27,28^, monolayer of group-V binary compounds^29^, fluorinated monolayer As and AsSb^30^, where the ZA branch (out of plane acoustical modes) becomes soft and get imaginary frequencies near the Γ point. It is believed such instability can be removed by defects, such as ripples, or finite size sheets. On the other hand, the ZA imaginary frequencies around Γ point also depends on the mesh size used in the calculations. It may be an artifact of the mesh size since the interatomic forces related with ZA modes decay rapidly. One way to get rid of them is to use a finer mesh grid in the DFT calculation. Therefore, SnCH_3_ film is also dynamically stable. Generally, the buckled configuration can endure a larger mechanical distortion than planar one. Hence the modulation of the structure and electronic properties can be realized by the strategy of external in-plane strain. For instance, a reasonable strain can induce topological phase transition in functionalized germanene^21,31^, group-IV and V monolayers^32–34^. To well understand the structural variation of SnCH_3_ film under a large strain without dissociation, the evolutions of the buckling height (*h*), Sn-Sn bond length (*d*) and buckling angle (*θ*) as a function of tensile strain are analyzed as plotted in Fig. 1(c). Under the tensile strain, the Sn-Sn bond length is changed slightly with respect to the variations of the buckling height and angle. For instance, under tensile strain of 6% the Sn-Sn bond length is only stretched by 4.5%, whereas the buckling height and angle are reduced by 11.7% and 15.9%, respectively. Consequently, the total energy is slightly increased as shown in Fig. 1(d). Within this tensile strain range the Sn-Sn bonds of SnCH_3_ film are covalently preserved.

**Figure 2:**
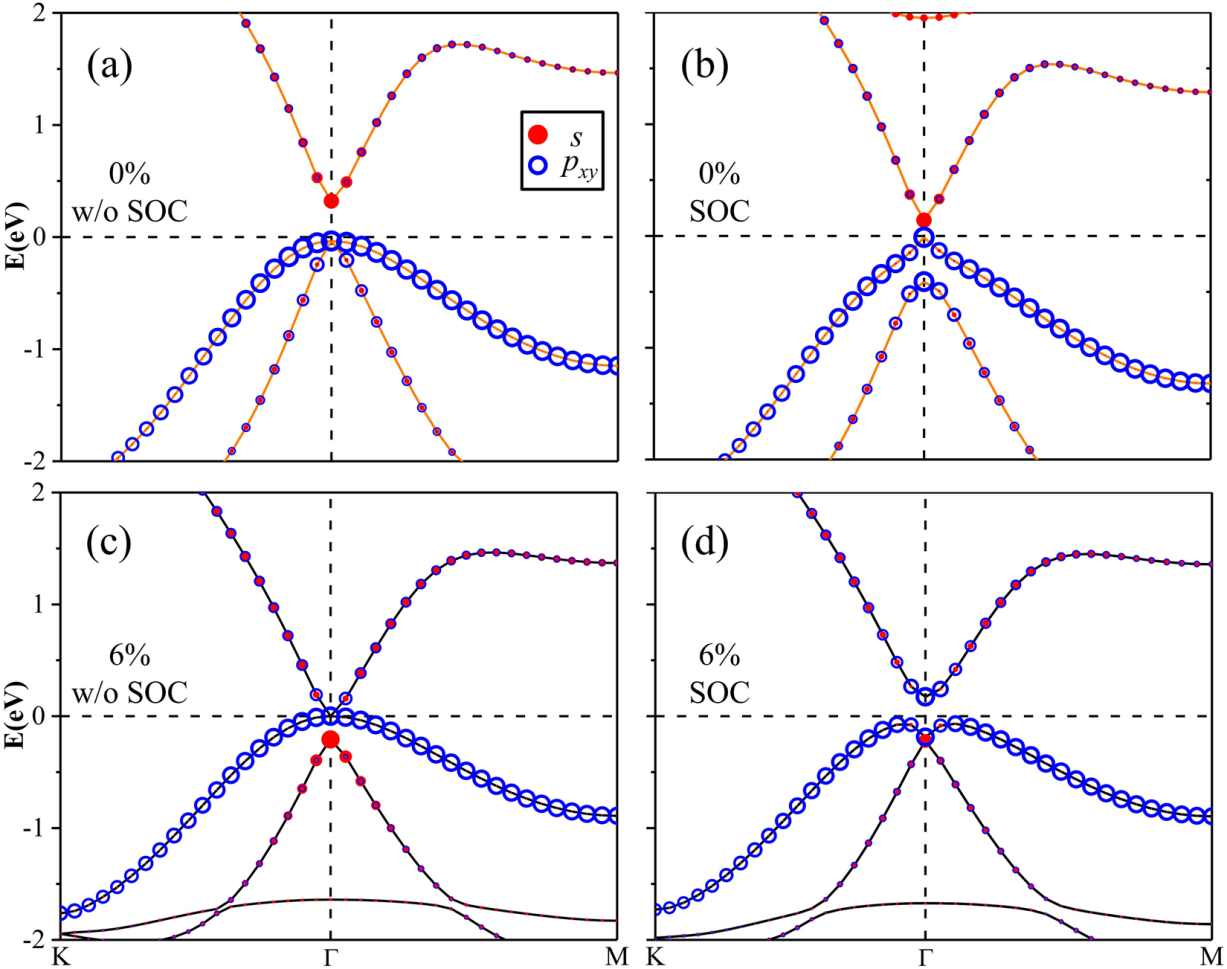
Band structures of SnCH_3_ film (**a**) without SOC and (**b**) with SOC under strain of 0%. Band structures of SnCH_3_ film (**c**) without SOC and (**d**) with SOC under strain of 6%. The radius of red dots and blue circles indicate the weight of *s* and *p*_*xy*_ orbitals. The Fermi level is set to zero.

Figure 2(a) displays the band structure of SnCH_3_ film at the equilibrium state without considering SOC. Compared with the case of pure stanene where two energy bands cross linearly at the K point^12^, a large band gap is substantially opened up at the K point by CH_3_ group decoration due to the saturation of the π orbital. Consequently, both the conduction band minimum (CBM) and valence band maximum (VBM) are shifted to the Γ point. Such similar feature is also observed for the halogenation of stanene. Significant differences between the two systems exist at the Γ point when excluding SOC. In SnCH_3_ film, a direct energy gap of about 0.356 eV appears with the valence and conduction bands separated at the Γ point. By projecting the bands onto different atomic orbitals, one can clearly see that the CBM is mainly occupied by *s* orbitals, whereas the twofold degenerate VBM is mostly dominated by *p*_*xy*_ orbitals. As we know, the SOC plays a key role in achieving QSH insulators. When the SOC is included, the band structure is calculated as shown in Fig. 2(b). We can find that the degenerate VBM is lifted out and split into two single states with the second valence band moved down. Moreover, the conduction bands are shifted downward close to Fermi level, leading to the band gap decreasing to 0.153 eV. From the partial orbital projection, the *s*-*p*_*xy*_-*p*_*xy*_ orbital order from top to bottom near the Fermi level at the Γ point is not varied by the SOC. This feature can also be found in hydrogenated stanene and GeCH_3_ films^35^, but different from halogenated stanene with *p*_*xy*_-*p*_*xy*_-*s* orbital order. It is quite clear that SnCH_3_ film belongs to trivial phase in topology, which is in good agreement with previous work^36^.

As the interval between *s* and *p*_*xy*_ energy levels at the Γ point sensitively depends on the Sn-Sn bonding strength, the band inversion and the associated QSH states can be effectively tuned by applying external strain. Here, we impose biaxial tensile strain on the SnCH_3_ film by turning the in-plane lattice parameter. The magnitude of strain is described by *ε* = (*a* − *a*_0_)/*a*_0_, where *a*_0_ and *a* denote the lattice constants of the unstrained and strained systems, respectively. With the increase of the tensile strain, the *s*-type CBM shifts down in energy and the band gap decreases. When the strain is about 6% as shown in Fig. 2(c), the SnCH_3_ film becomes gapless when excluding SOC. More interestingly, the so-called *s*-*p*-type band inversion takes place at the Γ point. The two *p*_*xy*_ bands touch each other at the Fermi level, whereas the *s* band moves down to the valence band region, resulting in *p*_*xy*_ − *p*_*xy*_ − *s* orbital order. Such band inversion is also driven in many other QSH insulators^12,21,31,37–39^. When we turn on the SOC, the band structure of the stretched SnCH_3_ film is plotted in Fig. 2(d). Unsurprisingly, the SOC opens a band gap in the gapless film. Furthermore, the VBM is transformed from the “Λ-shape” to “M-shape”. The direct band gap and indirect band gap are 0.359 eV and 0.240 eV, respectively. The above results show that the tensile strain drives the trivial SnCH_3_ film to a nontrivial QSH insulator. Such a large SOC gap is quite promising for achieving QSH states at room temperature. When the tensile strain is continually increased beyond 6%, the indirect band gap will be gradually enlarged. For instance, at the value of 12%, the energy gap reaches up to 0.262 eV. Within such strain range, the nontrivial topology feature is not damaged.

There are two strategies that have been widely used to confirm the topological nontrivial insulators. One is to calculate the topological invariant proposed by Fu and Kane^40^. For the 2D TI phase, the topological invariant is calculated from the parities of the Bloch wave functions for occupied bands at time-reversal invariant momenta (TRIM) points, one Γ and three M points, as

1 $${\delta }_{i}=\prod _{m=1}^{N}{\xi }_{2m}^{i}({K}_{i}),\quad {(-1)}^{\nu }=\prod _{i=1}^{4}{\delta }_{i}=\delta ({\rm{\Gamma }}){\delta }^{3}(M)$$

where *δ* is the product of parity eigenvalues at the TRIM points, *ξ* = ±1 denotes parity eigenvalues and *N* is the number of the occupied bands. According to the Z_2_ classification, *v* = 0 characterizes a trivial band topology, while *v* = 1 characterizes nontrivial phase. We calculate the parity eigenvalues of the Bloch wave function for the 11 occupied spin-degenerate bands at all TRIM points in stretched SnCH_3_ film, due to the preserve of structural inversion symmetry. As expected, in the equilibrium state without strain, the products of the parity eigenvalues at symmetry points: Γ and M are both +1, yielding a trivial topological invariant *v* = 0. With the strain increasing larger than 6.0%, the *s*-*p* band order exchanges at the Γ point. The parity eigenvalue of the VBM changes sign from +to −, while that at the M point remain +. Thus, the products of the parity eigenvalues at these two points are now distinct and the system becomes QSH insulator with *v* = 1.

**Figure 3:**
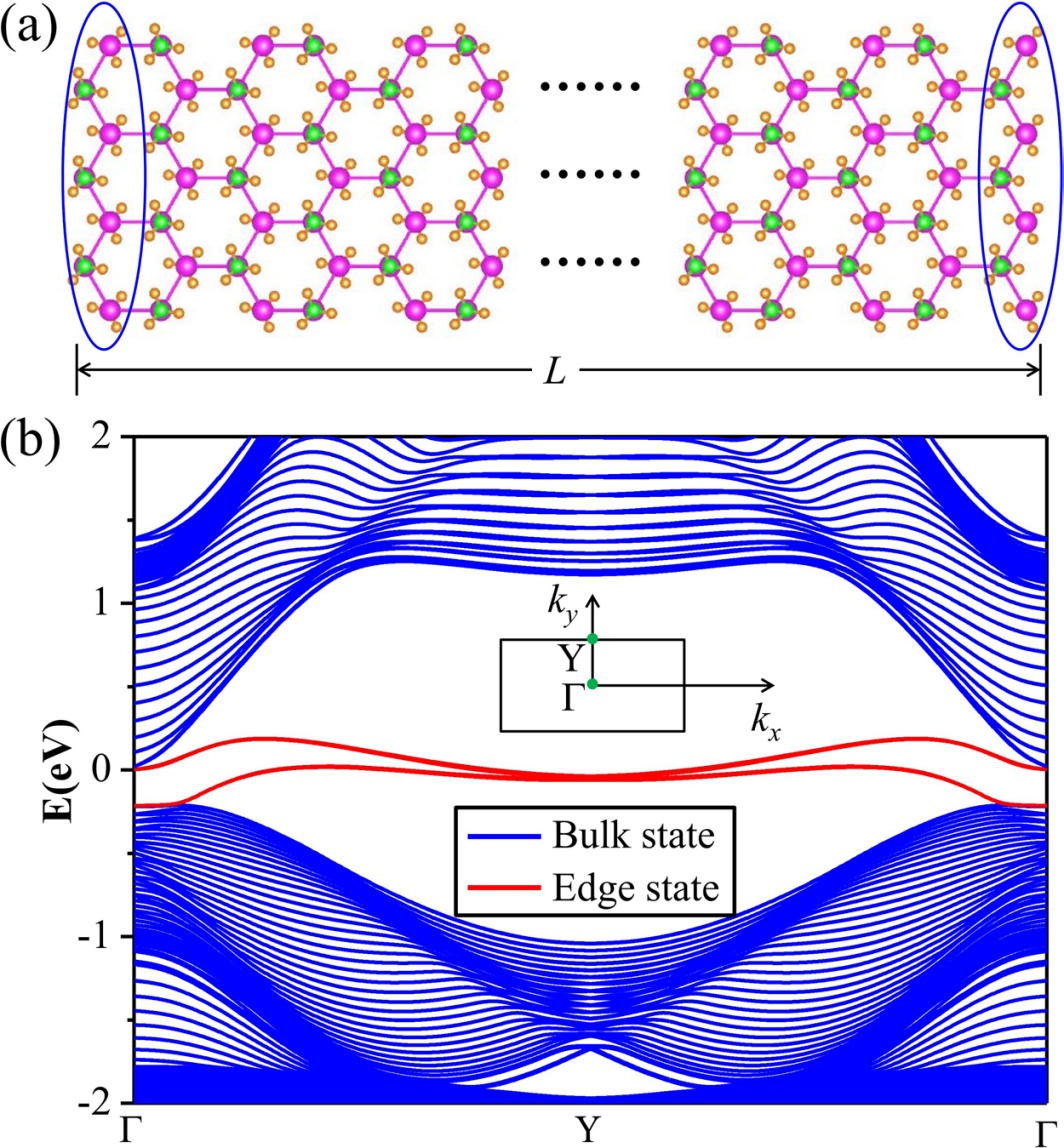
(**a**) Atomic structure of nanoribbon of SnCH_3_ with zigzag edges. *L* is the width of nanoribbon. (**b**) Corresponding edge states of SnCH_3_ with 1D Brillouin zone indicated in the inset with Γ = 0 and Y = π/*L*. The Fermi level is set to zero.

Another method is to demonstrate the existence of helical gapless edge states in QSH insulators. In our work, we construct the zigzag-shaped-edges nanoribbon of SnCH_3_ under strain of 6% as shown in Fig. 3(a). The width of the nanoribbon is selected to be large enough to avoid interactions between the edge states. The band structure is presented in Fig. 3(b). One can clearly see that the bands within bulk gap that connect the conduction and valence bands, cross linearly at the Γ point, forming helical edge states. Such edge states are protected from elastic backscattering by time-reversal symmetry, which are of significance to be used in electronics and spintronics. All the above results consistently indicate that stretched SnCH_3_ film is an ideal candidate to realize QSH effect.

**Figure 4:**
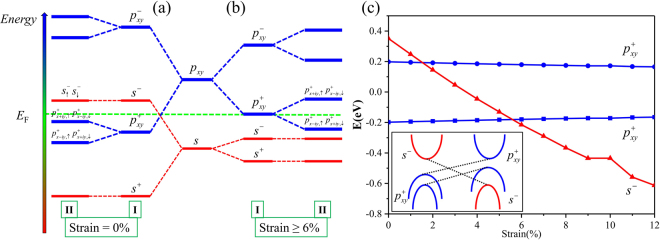
Schematic diagram of the evolution from the atomic *s* and *p*_*xy*_ orbitals into the conduction and valence bands at the Γ point for SnCH_3_ film (**a**) without strain and (**b**) with strain beyond 6%. The stages (I) and (II) represent the effect of switching on chemical bonding and SOC, respectively. The even and odd parities of Bloch states are denoted by +and −, respectively. (**c**) The relative energy levels of *s* and *p*_*xy*_ orbitals at the Γ point for SnCH_3_ film under different tensile strains, with a schematic representation shown in the inset. The center of the two separated *p*_*xy*_ levels is defined as zero in energy.

To physically understand the origin of the topological nature, here we start from atomic orbitals and consider the effect of chemical bonding and SOC on the energy levels at the Γ point for SnCH_3_ film as presented schematically in Fig. 4(a and b). The energy levels around the Fermi level are mainly composed of Sn-5*s* and Sn-5*p*_*xy*_ orbitals, where the *p*_*z*_ orbitals are saturated by CH_3_ group. According to the crystal field splitting theory in stage I, chemical bonding between Sn and Sn atoms makes the *s* and *p*_*xy*_ orbitals split into the bonding and anti-bonding states, labeled as *s* ^±^ and ${p}_{xy}^{\pm }$, swhere the superscript (+,−) denotes the parities of corresponding Bloch states, respectively. In the equilibrium state, the bands close to the Fermi level are contributed by the *s* ^−^ and ${p}_{xy}^{+}$, with *s* ^−^ being above the ${p}_{xy}^{+}$ in energy as shown in Fig. 4(a). Before turning on SOC, the ${p}_{xy}^{+}$ orbitals are degenerate, and the system is a semiconductor. When the SOC is taken into account in stage II, the degeneracy of the ${p}_{xy}^{+}$ levels is lifted, and the band gap is decreased. When considering the tensile strain as shown in Fig. 4(b), the interaction between the Sn atoms weakens because of the enlarged lattice constant, which makes the splitting between the bonding and anti-bonding states decreased, with *s* ^−^ level shifted down and ${p}_{xy}^{+}$ shifted up. Therefore, the electronic structure can be continuously tuned with strain increased, and the order of *s* ^−^ and ${p}_{xy}^{+}$ is inverted at critical point of 6.0%. The *s* ^−^ level becomes fully occupied, whereas the quadruply degenerate ${p}_{xy}^{+}$ is half occupied. Consequently the Fermi level stays at ${p}_{xy}^{+}$ level, exhibiting a zero-gap semiconductor. As the SOC effect is turn on, the degeneracy of the ${p}_{xy}^{+}$ orbital is lifted, opening a larger energy gap. To illustrate the band inversion process explicitly, we illustrate the *s*-*p* band inversion diagram in Fig. 4(c). With the tensile strain increased, the antibonding state *s* ^−^ shifts downward with respect to the bonding state ${p}_{xy}^{+}$. A crossing between the *s* ^−^ and ${p}_{xy}^{+}$ level occurs at ~6%, which leads to a parity exchange between occupied and unoccupied bands. therefore, a topological phase transition from a trivial insulator to a nontrivial phase is induced. The mechanism is similar as for HgTe quantum well^4^, but different from the nontrivial topology in the previous works^31–44^, which originates from the massive Dirac cone, and there is no band inversion.

Since standard PBE calculation usually underestimates the band gap, we adopt the more reliable hybrid functional HSE06 (see Supplementary Figure S1). The results in Fig. S1(a) and (b) show that the band gap of SnCH_3_ film without SOC is 0.837 eV with no strain, while the band gap with SOC decreases to 0.609 eV with the bands split. The band inversion associated QSH states occurs when the stretched strain is up to 9%, where the nontrivial indirect band gap is 0.314 eV as shown in Fig. S1(d), larger than that by PBE calculation. In addition, we find that as the stanene thickness increases (2–4 BLs), the topological phase transition from trivial to nontrivial appears compared to methyl-decorated 1 BL films (see Supplementary Figures S2 and S3). Figure S2 shows the atomic structure of methyl-decorated 4 BL stanene film seen from top and side. Equilibrium lattice constants for the methyl-decorated 2–4 BL stanene films are all 4.71 Å, quite close to the bulk value of 4.716 Å. The band structures in Fig. S3 show that 2 and 3 BL films are both insulator, while 4 BL film is semimetal with negative indirect energy gap. To examine their topology, the Z_2_ invariants are calculated for 2–4 BL films. The results of parity analysis at the four time-reversal invariant symmetry points show that the Z_2_ invariants in these films are all 1. Therefore, 2 and 3 BL films are nontrivial insulator, while 4 BL film is a nontrivial semimetal. In order to gain insight into the nature of band inversion in the films, we investigated orbital-projected band structures with SOC. Our analysis shows that the band inversion involved in the methyl-decorated 2–4 BL stanene films is of *s*-*p* type.

**Figure 5:**
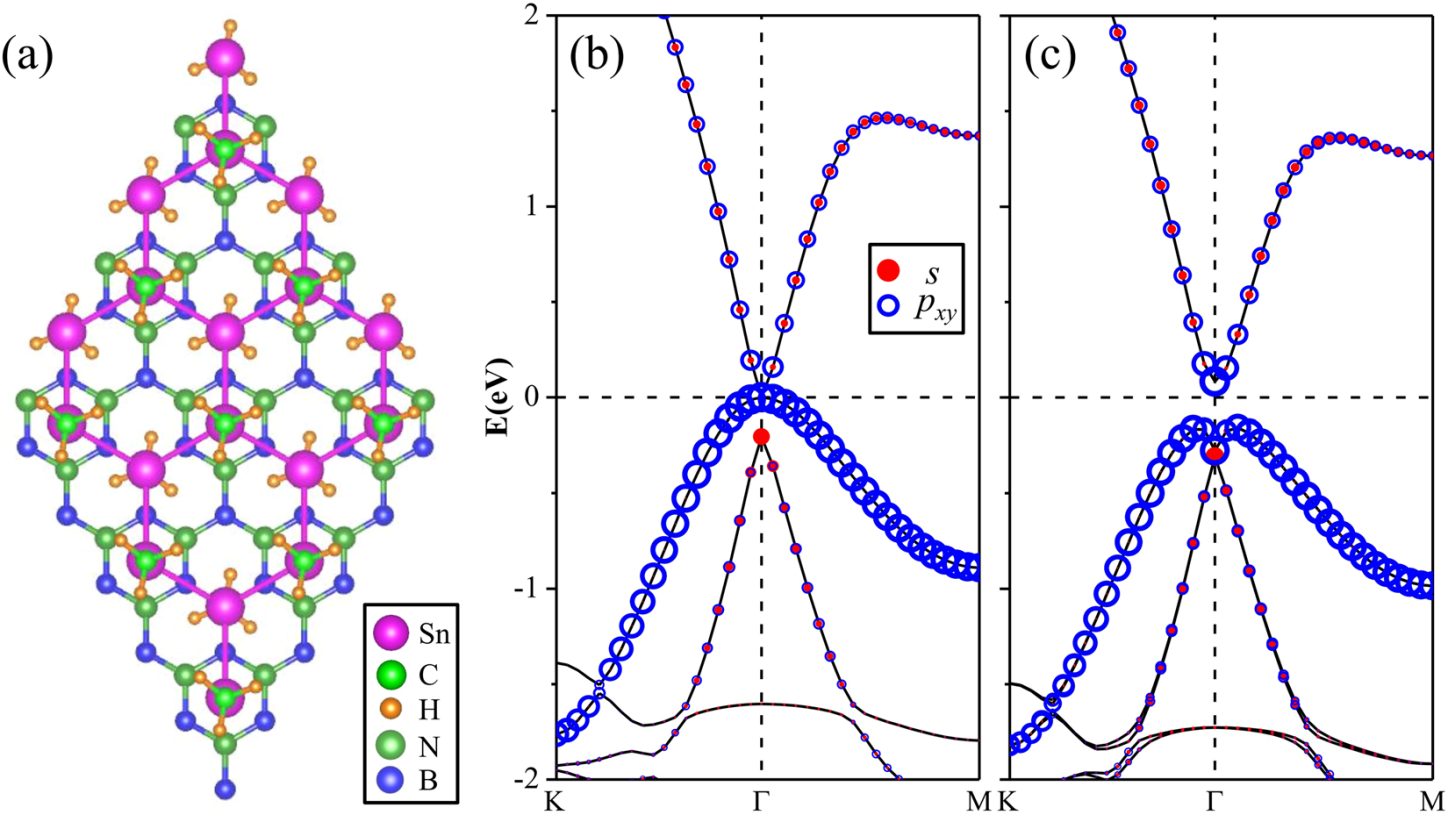
(**a**) Top view of atomic structure of 6%-strained SnCH_3_ on *h*-BN substrate. Corresponding band structures of 6%-strained SnCH_3_/*h*-BN heterostructure (**b**) without SOC and (**c**) with SOC. The radius of red dots and blue circles indicate the weight of *s* and *p*_*xy*_ orbitals. The Fermi level is set to zero.

To be well applied in electronic devices, the proper substrate materials are indispensable to support the nontrivial topology. Previous works indicate that the topologically insulating properties of silicene and germanene are easily destroyed by the substrates^45–47^, due to the lattice mismatch and interaction with substrates. Because the band inversion takes place at the Γ point rather than K point, the nontrivial nature in SnCH_3_ would be quite robust when they are on the substrate. Furthermore, the full saturation of *p*_*z*_ orbitals of Sn atoms ensures a weak interaction between SnCH_3_ with the substrate. Given these factors, we take 1 BL stretched SnCH_3_ film with strain of 6% for example where the film are placed on top of a 2 × 2 hexagonal BN (*h*-BN) substrate forming SnCH_3_/*h*-BN heterostructure, as shown in Fig. 5(a). In this case, lattice mismatch between SnCH_3_ (5.0244 Å for *ε* = 6%) and *h*-BN substrate (5.02 Å for 2 × 2 supercell) is extremely small. To correctly describe the van der Waals interaction, we use a DFT-D2 method of Grimme^48^, which has been demonstrated to reliably describe 2D heterostructures. The optimized interlayer spacing between adjacent layers is about 2.824 Å. The binding energy is obtained to be 0.204 eV per unit cell, indicating a weak interaction between SnCH_3_ and BN sheet. The band structures without and with the SOC are presented in Fig. 5(b and c), respectively. One can see that when excluding the SOC, the *p*_*xy*_ type bands mainly contributed by SnCH_3_ film are degenerate to form Dirac point at the Fermi level at the Γ point. The SOC band gap opened at the Dirac point and the nontrivial bands are intact in comparison to that in Fig. 2(d). These results demonstrate that it is feasible to deposit SnCH_3_ film on the *h*-BN substrate so as to attain QSH states.

Finally, we want to point out that the SnCH_3_ film is more promising than GeCH_3_ film to realize QSH effect at room temperature, since stretched SnCH_3_ film possesses a larger nontrivial energy gap. Moreover, the critical strain applied for SnCH_3_ film is much smaller than that for GeCH_3_ to induce topological phase transition, which is more flexible to carry out in experiment. Such difference can be construed as that topological property is closely related to the Sn-Sn or Ge-Ge bond lengths. Although GeCH_3_ and SnCH_3_ film shares similar geometric structure, the Ge-Ge bond lengths are relatively less than those Sn-Sn bonds due to the strong bonding. The difference between *s* and *p*_*xy*_ orbitals energy level thus is very large, resulting in a more sizable band gap when excluding the SOC. Therefore, much larger critical tensile strain is needed to drive band inversion in GeCH_3_ film. In view of experimental realization, the similar strategy that biaxial strain larger than 10% has been achieved in graphene^49^, may be helpful for SnCH_3_ film.

In summary, based on the first-principles calculations, we proposed that SnCH_3_ film can be tuned to a quantum spin Hall insulator by imposing biaxial tensile strain larger than 6%. The topological phase transition from a trivial to nontrivial insulator can be ascribed to the strain-induced *s*-*p*_*xy*_ type band inversion at the Γ point. The role of the SOC in stretched SnCH_3_ is to open up a relatively large energy gap. The value of band gap is about 0.240 eV at critical strain of 6%, which increases with continual increase of tensile strain, while the band gap using more reliable hybrid functional HSE06 is increase to 0.314 eV. These interesting results make SnCH_3_ film a promising candidate to achieve quantum spin Hall effect at room temperature, which is significant for future electronic devices with low-power consumption.

## Electronic supplementary material

Supplementary Information

## References

[1] Kane CL, Mele EJ (2005). Quantum spin Hall effect in grapheme. Phys. Rev. Lett..

[2] Kane CL, Mele EJ (2005). Z_2_ topological order and the quantum spin Hall effect. Phys. Rev. Lett..

[3] Bernevig BA, Zhang S-C (2006). Quantum spin Hall effect. Phys. Rev. Lett..

[4] Bernevig BA, Hughes TL, Zhang S-C (2006). Quantum spin Hall effect and topological phase transition in HgTe quantum wells. Science.

[5] Qi XL, Zhang S-C (2010). The quantum spin Hall effect and topological insulators. Phys. Today.

[6] Hasan MZ, Kane CL (2010). Colloquium: Topological insulators. Rev. Mod. Phys..

[7] Novoselov KS (2004). Electric field effect in atomically thin carbon films. Science.

[8] Cahangirov S, Topsakal M, Aktürk E, Sahin HS, Ciraci S (2009). Two- and one-dimensional honeycomb structures of silicon and germanium. Phys. Rev. Lett..

[9] Fleurence A (2012). Experimental evidence for epitaxial silicene on diboride thin films. Phys. Rev. Lett..

[10] Bianco E (2013). Stability and exfoliation of germanane: a germanium graphane analogue. ACS Nano..

[11] Liu CC, Feng W, Yao YG (2011). Quantum spin Hall effect in silicene and two-dimensional germanium. Phys. Rev. Lett..

[12] Xu Y (2013). Large-gap quantum spin Hall insulators in tin films. Phys. Rev. Lett..

[13] Fang YM (2015). Quantum Spin Hall States in Stanene/Ge(111). Sci Rep..

[14] Zhu FF (2015). Epitaxial growth of two-dimensional stanine. Nat. Mater..

[15] Zhao J, Lia YL, Ma J (2016). Quantum spin Hall insulators in functionalized arsenene (AsX, X = F, OH and CH_3_) monolayers with pronounced light absorption. Nanoscale.

[16] Li SS (2016). Robust Room-Temperature Quantum Spin Hall Effect in Methyl-functionalized InBi honeycomb film. Sci Rep..

[17] Ma YD (2015). Two-dimensional inversion-asymmetric topological insulators in functionalized III-Bi bilayers. Phys. Rev. B.

[18] Crisostomo CP (2015). Robust Large Gap Two-Dimensional Topological Insulators in Hydrogenated III-V Buckled Honeycombs. Nano Lett..

[19] Yao LZ (2015). Predicted Growth of Two-Dimensional Topological Insulator Thin Films of III-V Compounds on Si(111) Substrate. Sci. Rep..

[20] Jiang SS (2014). Improving the stability and optical properties of germanane via one-step covalent methyl-termination. Nat. Commun..

[21] Ma YD, Dai Y, Wei W, Huang BB, Whangbo MH (2014). Strain-induced quantum spin Hall effect in methyl-substituted germanane GeCH_3_. Sci. Rep..

[22] Kresse G, Furthmuller J (1996). Efficient iterative schemes for ab initio total-energy calculations using a plane-wave basis set. Phys. Rev. B.

[23] Kresse G, Joubert D (1999). From ultrasoft pseudopotentials to the projector augmented-wave method. Phys. Rev. B.

[24] Kresse G, Furthmuller J (1996). Efficiency of ab-initio total energy calculations for metals and semiconductors using a plane-wave basis set. Comput. Mater. Sci..

[25] Perdew JP, Burke K (1996). Generalized gradient approximation made simple. Phys. Rev. Lett..

[26] Togo A, Oba F, Tanaka I (2008). First-principles calculations of the ferroelastic transition between rutile-type and CaCl_2_-type SiO_2_ at high pressures. Phys. Rev. B.

[27] Şahin H (2009). Monolayer honeycomb structures of group-IV elements and III-V binary compounds: first-principles calculations. Phys. Rev. B.

[28] Tang PZ (2014). Stable two-dimensional dumbbell stanene: a quantum spin Hall insulator. Phys. Rev. B.

[29] Nie Y (2015). Strain induced topological phase transitions in monolayer honeycomb structures of group-V binary compounds. Sci. Rep..

[30] Zhang QY, Schwingenschlögl U (2016). Emergence of Dirac and quantum spin Hall states in fluorinated monolayer As and AsSb. Phys. Rev. B.

[31] Si C (2014). Functionalized germanene as a prototype of large-gap two-dimensional topological insulators. Phys. Rev. B.

[32] Zhao MW, Zhang XM, Li LY (2015). Strain-driven band inversion and topological aspects in Antimonene. Sci. Rep..

[33] Zhang HJ, Ma YD, Chen ZF (2015). Quantum spin hall insulators in strain-modified arsenene. Nanoscale.

[34] Huang ZQ (2014). Strain driven topological phase transitions in atomically thin films of group IV and V elements in the honeycomb structures. New J. Phys..

[35] Chou BH (2014). Hydrogenated ultra-thin tin films predicted as two dimensional topological insulators. New J. Phys..

[36] Ma YD, Dai Y, Kou LZ, Frauenheim T, Heine T (2015). Robust two-dimensional topological insulators in methyl-functionalized bismuth, antimony, and lead bilayer films. Nano Lett..

[37] Zhao MW, Chen X, Li LY, Zhang XM (2015). Driving a GaAs film to a large-gap topological insulator by tensile strain. Sci. Rep..

[38] Yan BH, Müchler L, Felser C (2012). Prediction of weak topological insulators in layered semiconductors. Phys. Rev. Lett..

[39] Wang AZ, Du AJ, Zhao MW (2015). Prediction of a large-gap quantum-spin-Hall insulator: Diamond-like GaBi bilayer. Nano Res..

[40] Fu L, Kane CL (2007). Topological insulators with inversion symmetry. Phys. Rev. B.

[41] Wang DC (2015). Topological states modulation of Bi and Sb thin films by atomic adsorption. Phys. Chem. Chem. Phys..

[42] Wang DC (2016). Robust large-gap quantum spin Hall insulators in chemically decorated arsenene films. New J. Phys..

[43] Wang DC (2016). Quantum spin Hall insulator in halogenated arsenene films with sizable energy gaps. Sci. Rep..

[44] Ma YD, Kou LZ, Du AJ, Heine T (2015). Group 14 element-based non-centrosymmetric quantum spin Hall insulators with large bulk gap. Nano Res..

[45] Vogt P (2012). Silicene: compelling experimental evidence for graphenelike two-dimensional silicon. Phys. Rev. Lett..

[46] Ni ZY (2012). Tunable bandgap in silicene and germanene. Nano Lett..

[47] Li LF (2014). Buckled germanene formation on Pt (111). Adv. Mater..

[48] Bučko T, Hafner J, Lebèfue S, Ángyán JG (2010). Improved Description of the Structure of Molecular and Layered Crystals: Ab Initio DFT Calculations with van der Waals Corrections. J. Phys. Chem. A.

[49] Shioya H, Craciun MF, Russo S, Yamamoto M, Tarucha S (2014). Straining graphene using thin film shrinkage methods. Nano Lett..

