# Supplementary material for: Strain induced band inversion and topological phase transition in methyl-decorated stanene film
Source: Sci Rep. 2017 Dec 6;7:17089. doi: 10.1038/s41598-017-17336-8 (PMC5719061; doi:10.1038/s41598-017-17336-8)
Supplement: Supplementary file 1 — Supplementary Information [file 41598_2017_17336_MOESM1_ESM.doc]

**Supplementary Information**

**Strain induced band inversion and topological phase transition in** **methyl-decorated stanene film**

Dongchao Wang^1^, Li Chen*^1^, Hongmei Liu^1^, Changmin Shi^1^, Xiaoli Wang^1^, Guangliang Cui^1^, Pinhua Zhang^1^ and Yeqing Chen^1^

*^1^Institute of Condensed Matter Physics, Linyi University, Shandong 276000, China*

*Corresponding author: chenli@lyu.edu.cn


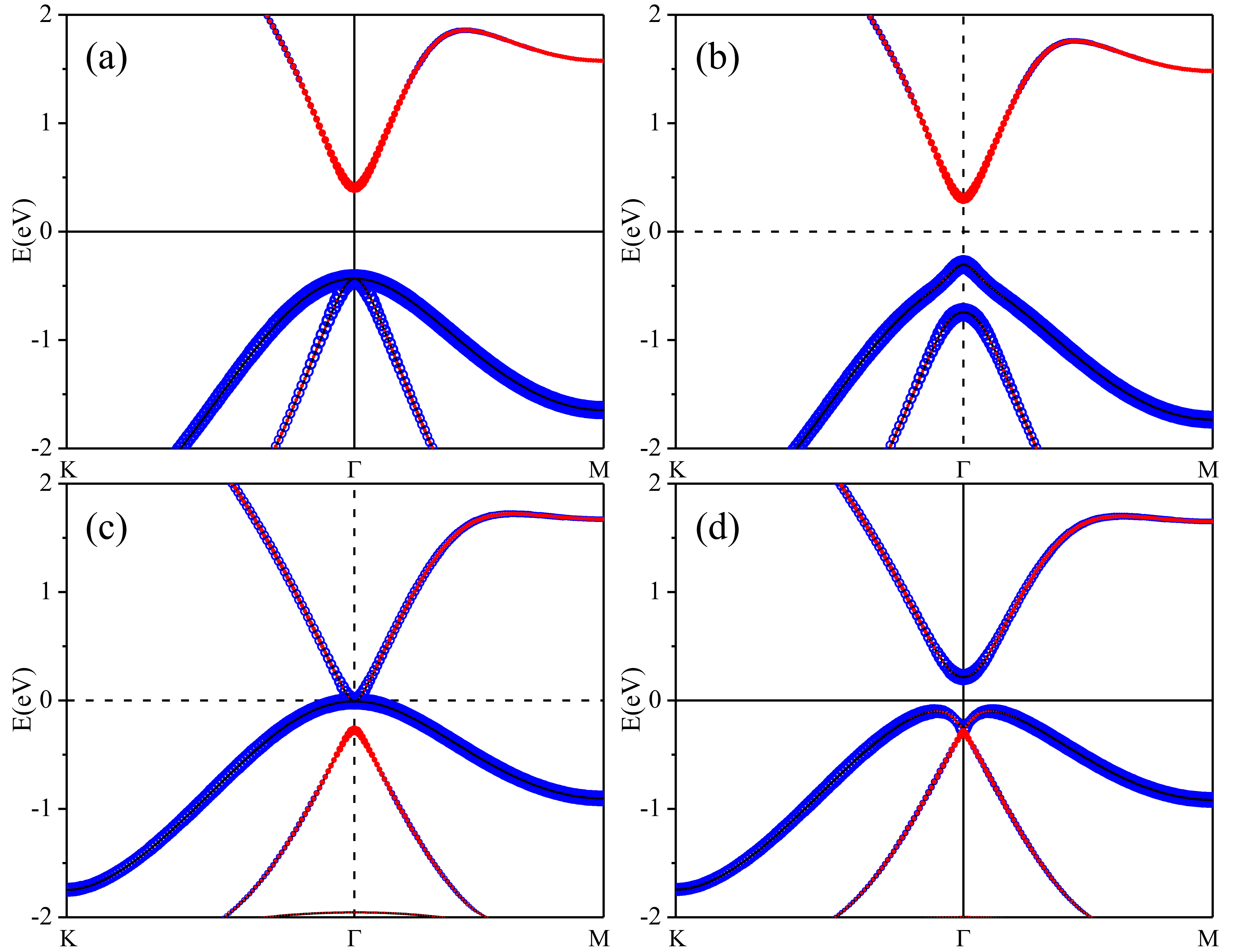


**Figure S1.** Band structures calculated by using hybrid functional HSE06. (a)-(b) Band structures of SnCH_3_ film without and with SOC under strain of 0%. (c) and (d) Band structures without and with SOC under strain of 9%. The radius of red dots and blue circles indicate the weight of *s* and *p_xy_* orbitals. The Fermi level is set to zero.

Since standard PBE calculation usually underestimates the band gap, we adopt the more reliable hybrid functional HSE06 (see Supplementary Figure S1). The results in Fig. S1(a) and (b) show that the band gap of SnCH_3_ film without SOC is 0.837 eV with no strain, while the band gap with SOC decreases to 0.609 eV with the bands split. The band inversion associated QSH states occurs when the stretched strain is up to 9%, where the nontrivial indirect band gap is 0.314 eV as shown in Fig. S1(d), larger than that by PBE calculation.


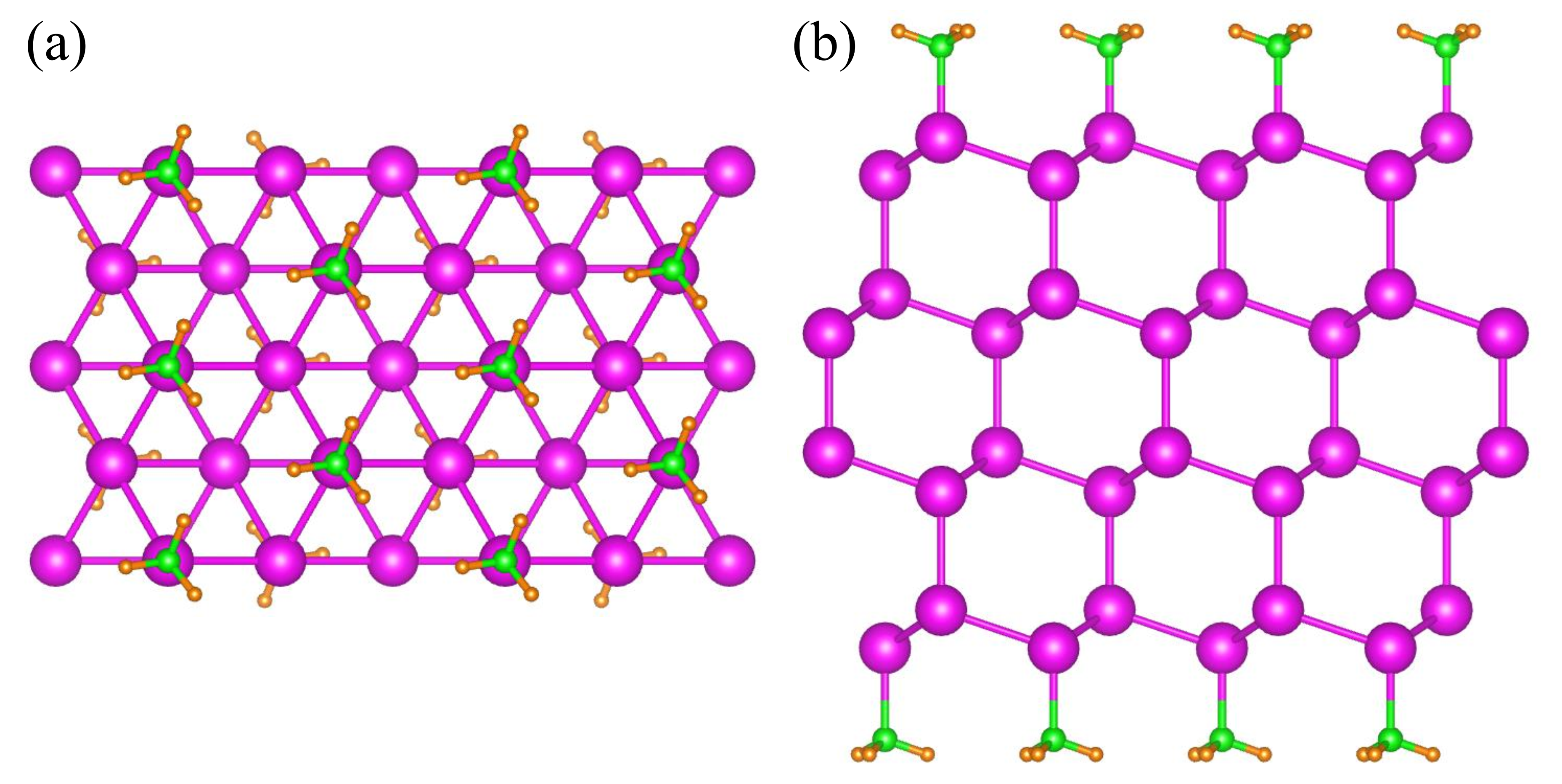


Figure S2. (a) and (b) Top and side views of atomic structure of methyl-decorated 4 BL stanene film.


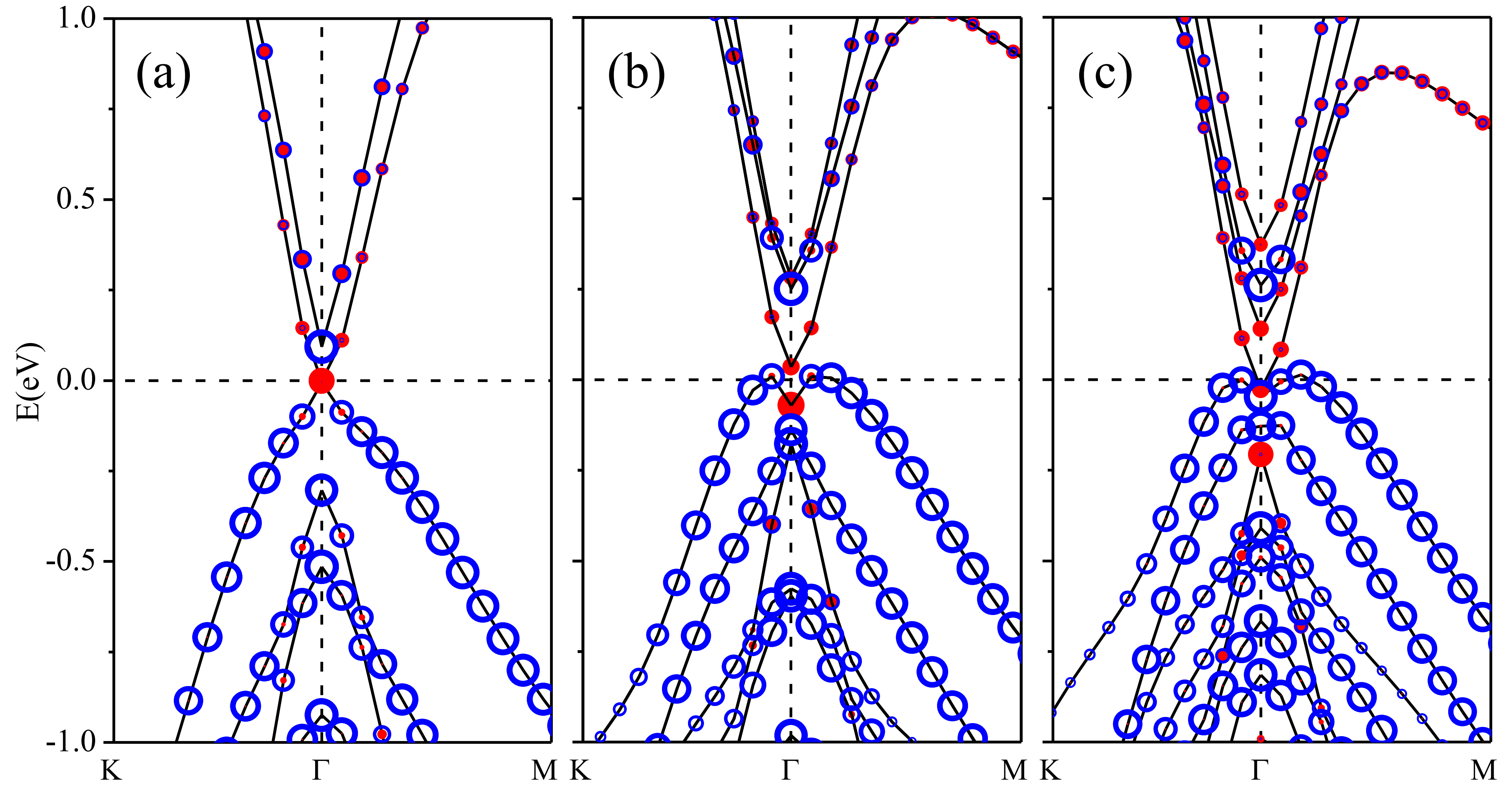


Figure S3. (a)-(c) Band structures of methyl-decorated 2-4 BL stanene films with SOC, respectively. The radius of red dots and blue circles indicate the weight of *s* and *p_xy_* orbitals. The Fermi level is set to zero.

Figure S2 shows the atomic structure of methyl-decorated 4 BL stanene film seen from top and side. Equilibrium lattice constants for the methyl-decorated 2-4 BL stanene films are all 4.71 Å, quite close to the bulk value of 4.716 Å. The band structures in Fig. S3 show that 2 and 3 BL films are both insulator, while 4 BL film is semimetal with negative indirect energy gap. To examine their topology, the Z_2_ invariants are calculated for 2-4 BL films. The results of parity analysis at the four time-reversal invariant symmetry points show that the Z_2_ invariants in these films are all 1. Therefore, 2 and 3 BL films are nontrivial insulator, while 4 BL film is a nontrivial semimetal. In order to gain insight into the nature of band inversion in the films, we investigated orbital-projected band structures with SOC. Our analysis shows that the band inversion involved in the methyl-decorated 2-4 BL stanene films is of *s*-*p* type.
